# Multi-class sentiment analysis of urdu text using multilingual BERT

**DOI:** 10.1038/s41598-022-09381-9

**Published:** 2022-03-31

**Authors:** Lal Khan, Ammar Amjad, Noman Ashraf, Hsien-Tsung Chang

**Affiliations:** 1 Department of Computer Science and Information Engineering, Chang Gung University, Taoyuan, Taiwan; 2 CIC, Instituto Politécnico Nacional, Mexico City, Mexico; 3 Department of Physical Medicine and Rehabilitation, Chang Gung Memorial Hospital, Taoyuan, Taiwan; 4 Artificial Intelligence Research Center, Chang Gung University, Taoyuan, Taiwan; 5 Bachelor Program in Artificial Intelligence, Chang Gung University, Taoyuan, Taiwan

**Keywords:** Machine learning, Computer science, Information technology, Computational science

## Abstract

Sentiment analysis (SA) is an important task because of its vital role in analyzing people’s opinions. However, existing research is solely based on the English language with limited work on low-resource languages. This study introduced a new multi-class Urdu dataset based on user reviews for sentiment analysis. This dataset is gathered from various domains such as food and beverages, movies and plays, software and apps, politics, and sports. Our proposed dataset contains 9312 reviews manually annotated by human experts into three classes: positive, negative and neutral. The main goal of this research study is to create a manually annotated dataset for Urdu sentiment analysis and to set baseline results using rule-based, machine learning (SVM, NB, Adabbost, MLP, LR and RF) and deep learning (CNN-1D, LSTM, Bi-LSTM, GRU and Bi-GRU) techniques. Additionally, we fine-tuned Multilingual BERT(mBERT) for Urdu sentiment analysis. We used four text representations: word *n*-grams, char *n*-grams,pre-trained fastText and BERT word embeddings to train our classifiers. We trained these models on two different datasets for evaluation purposes. Finding shows that the proposed mBERT model with BERT pre-trained word embeddings outperformed deep learning, machine learning and rule-based classifiers and achieved an F1 score of 81.49%.

## Introduction

Social networks (SNs) such as Blogs, Forums, Facebook, YouTube, Twitter, Instagram, and others have recently emerged as the most important platforms for social communication between diverse people^1,2^. As technology and awareness grow, more people are using the internet for global communication, online shopping, sharing their experiences and thoughts, remote education, and correspondence on numerous aspects of life^3–5^. Users are increasingly using SNs to communicate their views, opinions, and thoughts, as well as participate in discussion groups^6^. The inconspicuousness of the World Wide Web (WWW) has permitted single user to engage in aggressive SNs speech data that has made text conversation^7,8^ or, more precisely, sentiment analysis (SA) is vital to understand the behaviors of people^9–15^.

The significance of sentiment analysis may be seen in our desire to know what they think and how others feel about the problem^16^. Firms and governments are looking for useful information in these user comments such as the feelings behind client comments^17^. SA refers to the application of machine and deep learning and computational linguistics to investigate the feelings or views expressed in user-written comments^18,19^. Because of increasing interest in SA, businesses are interested in driving campaigns, having more clients, overcoming their weaknesses, and winning marketing tactics. Business firms are interested to know the individual’s feedback and sentiments about their product and services^20^. Furthermore, politicians and their political parties are interested in learning about their public reputations. Due to the recent surge in SNs, sentiment analysis focus has shifted to social media data research. The importance of SA has increased in several fields, including movies, plays, sports, news chat shows, politics, harassment, services, and medical^21^. SA includes enhanced techniques for NLP, data mining for predictive studies, and topic modeling becomes an exciting domain of research^22^.

In terms of linguistics and technology, English and particular other European dialects are recognized as rich dialects. Yet, many other languages are classified as resource-deprived^23^, Urdu is one of them. The Urdu language requires a standard dataset, but unfortunately, scholars face a shortage of language resources. The Urdu language is Pakistan’s national and one of the official languages spoken in some state and union territories of India.

Sentiment analysis is as important for Urdu dialects as it is for any other dialect. Many obstacles make SA of the Urdu language difficult such as Urdu contains both formal and informal verb forms as well as masculine and feminine genders for each noun. Similarly, the Persian, Arabic, and Sanskrit languages have their terms in Urdu. Urdu is written from right to left, and the distinction between words is not always clear. The scarcity of acknowledged lexical resources^24,25^ and the lack of Urdu text data due to morphological concerns. Rather than a conventional text encoding scheme, most Urdu websites are organized in an illustrated manner, which complicates the task of producing a state-of-the-art machine-readable corpus. The well-known sentiment lexicon database is an essential component for constructing sentiment analysis classification applications in any dialect. SentiWordNet is one of the several sentiment lexicons available in English. Urdu, on the other hand, is a resource-poor language with a severe lack of sentiment lexicon. Problems with Urdu word segmentation, morphological structure and vocabulary variances are among the main deterrents to developing a fully effective Urdu sentiment analysis model.

### Research objective

This research aims to classify the semantic orientation of Urdu reviews. Our purposed model is inspired by^26^. In the cited paper, sentiment analysis of Arabic text was performed using pre-trained word embeddings. Recently, pre-trained algorithms have shown the state of the art results on NLP-related tasks^27–30^. These pre-trained models are trained on large corpus in order to capture long-term semantic dependencies.

The objective of this research study is to answer the following questions:

- Is it possible to utilize a deep learning model in combination with a pre-trained word embedding strategy to identify the sentiment expressed by a social network user in Urdu?
- Does the deep learning approach with fastText and BERT word embedding effective than the machine learning-based approaches and the rule-based approach to sentiment analysis for the Urdu language that have been studied so far?

To answer the first study question, the use of pre-trained word embeddings for sentiment analysis of Urdu language reviews is investigated. A deep learning model based on pre-trained word embedding captures long-term semantic relationships between words, unlike rule-based and machine learning-based approaches. To answer the second question, the deep learning models were compared to the machine learning-based methods and the rule-based method of Urdu sentiment analysis.

The main contribution of our research are as follows:

- A new Multi-class sentiment analysis dataset for Urdu language based on user reviews. It is gathered from various domains such as food and beverages, movies and plays, software and apps, politics and sports. To the best of our knowledge, no such public Urdu corpus exists. The corpus will be made publicly available.
- Fine-tuning a multilingual BERT model for Urdu sentiment classification, which has been trained on 104 languages, including Urdu, and is based on a BERT base with 12 layers, 768 hidden heads, and 110M parameters.
- A set of baseline results of rule-based approach, machine-learning models (LR, MLP, Ada-Boost, RF, SVM) and deep learning models (1D-CNN, LSTM, Bi-LSTM, GRU and Bi-GRU) to create a benchmark for multi-class sentiment analysis using different text representations: fastText pre-trained word embeddings, char *n*-gram and word *n*-gram features.

The rest of the paper is organized as follows. Section “Related work” explains the related work for sentiment analysis. Section “Corpus generation” describes the creation of dataset and its statistics. Section “Proposed methodology” presents the proposed methodology. Section “Results analysis” analyze the experimental results and evaluation measures. Section “Conclusion and implications” concludes the paper.

## Related work

In this section, we give a quick overview of existing datasets and popular techniques for sentiment analysis.

### Sentiment analysis datasets

SemEval challenges are the most prominent efforts taken in the existing literature to create standard datasets for SA. In each competition, scholars accomplish different tasks to examine semantic analysis classifications using different corpora. The outcome of such competitions is a group of standard datasets and diverse approaches for SA. These benchmark corpora have been created in the English and Arabic languages^31^. Mainly, user tweets/reviews belong to various genres such as hotel, restaurants and laptops.

Every time, the SemEval contests series comes up with the various size of corpora. In the 2013 edition, the SemEval competition used SMS and Twitter corpora, and the Twitter corpus contains a total of 15,195 reviews, was split into training, development, and testing data are 9728, 1654, and 3813, respectively, while the SMS corpus consists of 2093 reviews was only used for testing purpose. The Twitter corpus comprises a total of 1853 reviews in the 2014 edition, including 86 sarcastic tweets for testing^32^. There were five separate subtasks in the 2016 and 2017 competition series. Each task’s corpus was divided into three sections: training, development, and testing. Subtask A, B, and D and subtask C and E sentences 30,632, 17,639, and 30,632 were used, respectively. There are 332 news articles in the Korean corpus for SA. Human experts manually annotated these news articles for sentiment analysis. The dataset contains 7713 subjectively annotated sentences and 17,615 opinionated expression tags utilizing the Korean Subjectivity Markup Language annotation method, reflecting the characteristics of Korean languages^33^.

Another corpus has been created in the Indonesian language. The Twitter streaming API was used to collect 3.5 million tweets^34^. A Roman Urdu corpus has been created, contains 10,021 user comments belonging to various domains such as politics, sports, food and recipes, software, and movies. All these sentences were manually annotated by three native speakers^35^.

### Methods for sentiment analysis

Several methods have been proposed in the existing literature to solve SA tasks, such as supervised and unsupervised machine learning. In SemEval 2014 competition, both Support Vector Machine (SVM) and rule-based machine learning methods were applied. The lexicons were utilized to find the sentiment polarities of reviews using the rule-based technique. The overall polarity of the review was computed by summing the polarity scores of all words in the review and dividing by their distance from the aspect term. If a sentence’s polarity score is less than zero (0), it is classified as negative; if the score is equal to zero, it is defined as neutral; and if the score is equal to or more than one, it is defined as positive. These classified features and *n*-gram features have been used to train machine learning algorithms. In SemEval 2016 contest edition, many machine learning algorithms such as Linear Regression (LR), Random Forest (RF), and Gaussian Regression (GR) were used^31^. The word embeddings are enhanced Natural Language Processing (NLP) method representing words or phrases into numerical numbers names as vector. Machine learning algorithms such as SVM will determine a hyperplane that classifies tweets/reviews according to their sentiment. Similarly, RF generates various decision trees, and each tree is examined before a final choice is made. In the same way, Nave Bayes (NB) is a probabilistic machine learning method that is based on the Bayes theorem^36^.

Many research studies have been published to execute SA of various resource-deprived dialects like as Khmer, Thai, Roman Urdu, Arabic and Hindi. Based on the negation and discourse relationship, a study on Hindi dialect has been conducted for sentiment analysis. A corpus of human-annotated reviews in Hindi was created. An accuracy of 80.21% was achieved using a polarity-based method^37^. Similarly, few research studies have been conducted in the Thai dialect, also considered resource-deprived languages^38^. Another study was carried out to identify abusive words in the Thai dialect. Eighty-six percent of the f-measure was attained using the machine learning method. Similarly, a research study has been conducted in the Bengali dialect^39^. In this study, the SA of Bengali reviews is executed using the word2vec embedding model. Results reveal that their proposed algorithm achieved an accuracy of 75.5%.

### Urdu datasets and machine learning techniques

The essential component of any sentiment analysis solution is a computer-readable benchmark corpus of consumer reviews. One of the most significant roadblocks for Urdu SA is a lack of resources, such as the lack of a gold-standard dataset of Urdu reviews. The truth is that most Urdu websites are designed in illustrative patterns rather than using standard Urdu encoding^40^. We recognized two methods for dataset creation from the existing literature, named as (1) automatic and (2) manual.

A research study focusing on Urdu sentiment analysis^41^ created two datasets of user reviews to examine the efficiency of the proposed model. Only 650 movie reviews are included in the C1 dataset, with each review averaging 264 words in length. There are 322 positive and 328 negative reviews in corpus C1. The other dataset named C2, contains 700 reviews about refrigerators, air conditions, and televisions. The average length of words per review is 196 words.

Another study^42^ used a corpus collected from the BBC Urdu news website to work on Urdu text classification. Two types of filters were successfully implemented to collect the required data. They concentrate on words like “Ghusa” (anger) and “Pyar” (love). A HTML parser is used to parse the obtained data, which yielded 500 news stories with 700 sentences containing the keywords mentioned above. These sentences were annotated for emotions. Nearly 6000 sentences not annotated with emotions were discarded from those 500 news articles.

Another study^43^ on Urdu sentiment analysis subjectivity developed a corpus consisting of 6025 sentences from151 Urdu blogs from 14 various domains. Three human specialists manually classified these comments into three categories: neutral, negative, and positive. Additionally, they have implemented five supervised machine learning algorithms like SVM, Lib, NB (KNN, IBK), PART, and decision tree. Results reveal that KNN achieves the highest accuracy of 67.01% and performs better than other supervised machine learning algorithms. However, the performance of models can be enhanced by increasing the corpus size and using deep learning methods with pre-trained word embedding models.

Similarly, in work^44^, the comparison of NB versus SVM for the language preprocessing steps of Urdu documents reveals that SVM performs better than NB regarding accuracy. Additionally, normalized term frequency gives much improved results for feature selection. The major drawback of the proposed system is that the tokenization is done based on punctuation marks and white spaces. However, due to the grammatical structure of the Urdu language, the writer may put white space between a single word such as (Khoubsorat, beautiful), which will cause the tokenizer to tokenize the single word as two words (khoub) and (sorat), which is incorrect.

According to this study^45^, authors used three classic machine learning algorithms, such as NB, SVM, and Decision tree followed by a supervised machine learning approach to create Word Sense Disambiguation (WSD) in Urdu text. They test their theories using a corpus generated from Urdu news websites. They attain an f-measure of 0.71%. However, by implanting an adaptive mechanism, the system’s accuracy could be increased.

### Urdu datasets and deep learning techniques

Deep learning approaches have recently been investigated for classification of Urdu text. In this study^46^, authors used deep learning methods to classify Urdu documents for product manufacturing. Stop words and infrequent words were deleted, which increased performance for medium and small datasets but decreased performance for large corpora. According to their findings, CNN with several filters (3,4,5) outperformed the competition, whereas BiLSTM outperformed CLSTM and LSTM. The authors of^47^ used a single layer CNN with several filters to classify documents at the document level, and the results outperformed the baseline approaches. For document classification^48^, compared the performance of hybrid, machine learning, and deep learning models. According to their findings, the normalized difference measure-based feature selection strategy increases the accuracies of all models.

In this study^49^, authors recently suggested a model for Urdu SA by examining deep learning methods along with various word embeddings. For sentiment analysis, the effectiveness of deep learning algorithms such as LSTM, BiLSTM-ATT, CNN, and CNN-LSTM was evaluated.

The most significant work^50^ has recently been performed on SA of Urdu text using various machine learning and deep learning techniques. Initially, Urdu user reviews of six various domains were collected from various social media platforms to build a state of art corpus. Later on, the whole Urdu corpus was manually annotated by human experts. Finally, a set of machine learning algorithms such as RF, NB, SVM, AdaBoost, MLP, LR, and deep learning algorithms such LSTM and CNN-1D were applied to validate the generated Urdu corpus. LR algorithms achieve the highest accuracy out of all others machine learning and deep learning algorithms.

A few research employing deep learning, semantic graphs and multimodal based system (MBS) have been undertaken on the areas of emotion classification^51^, concept extraction^52^, and user behavior analysis^53^. A unique CNN Text word2vec model was proposed in the research study^51^ to analyze emotion in microblog texts. According to the testing results the suggested MBS^52^ has a remarkable ability to learn the normal pattern of users’ everyday activities and detect anomalous behaviors.

**Table 1:** Summary of existing Urdu datasets.

Corpus	Publicly available	Classes	Algorithms	Acc (%)
6025 (various genres)^43^	Yes	3	SVM, Lib, NB, (KNN, IBK), PART and decision tree	67
650 (movies)^42^	No	2	Language prepossessing	40
700 (electronics appliances)^42^	No	2	Language prepossessing	38
26,057 documents^44^	No	–	NB and SVM for language prepossessing	–
Only 1000 opinions of Urdu news data^54^	No	3	Unsupervised (lexicon based)	86
9601 (various domain)^50^	Yes	2	Machine and deep learning	81
6000^49^	No	2	Deep learning	77.9
9312 reviews of various domains (proposed study)	Yes	3	Rule-based, deep learning and machine learning	78

There have been very few research studies on Urdu SA, and it is still in its early stages of maturation compared to other resource-rich languages like English. Because of the scarcity of linguistic resources, this can be discouraging for language engineering scholars. The majority of previous research papers^47^ focused on various areas of language processing such as stemming, stop word recognition and removal, and Urdu word segmentation and normalization. The summery of the existing literature is presented in Table 1.

Furthermore, the size of available annotated datasets is insufficient for successful sentiment analysis. However, the majority of the datasets and reviews from limited domains are only from negative and positive classes. To address this issue, this work focuses on the creation of an Urdu text corpus that includes sentences from several genres. To accomplish sentiment analysis task, we have applied various machine learning models with various features, deep learning models with combination of pre-trained word vectors and a rule-based algorithm on our created corpus UCSA-21 which has not yet investigated completely for the Urdu sentiment analysis text.

## Corpus generation

This section explains how a manually annotated Urdu dataset was created to achieve Urdu SA. The collection of user comments and reviews from multiple websites, the compilation of human annotation rules, the execution of manual annotation, standardization, and finally, the description of the dataset’s features are all phases involved in creating the Urdu Corpus for Sentiment Analysis (UCSA-21).

We gathered data from websites that offered unfettered access and allowed users to remark in Urdu to create a benchmark dataset for assessing Urdu sentiment. Table 2 summarizes all of the websites that we visited to get user reviews. Movies, Pakistani and Indian drama, TV discussion shows, food and recipes, politicians and Pakistani political parties, sport, software, blogs and forums and gadgets were among the genres from which we gathered data. During a 5- to 6-month period, three people who were well-versed in the objective manually collected user comments. Initially, the data was gathered into an excel sheet along with the following details: (1) the review ID; (2) the review’s domain; and (3) the annotation label.

**Table 2:** Online collection sources for Urdu user reviews.

Domain	Websites
Appliances, software and blogs	mobilesmspk.net, itforumpk.com, baazauq.blogspot.com, dufferistan.com, mbilalm.com, urduweb.org, urdudaan.blogspot.com, itdunya.com, achidosti.com, itdarasgah.com, tafrehmella.com, sachiidosti.com, urdupoint.com
Movies, news talk shows, and Pakistani and Indian dramas	Hamriweb.com, youtube.com, facebook.com, hamariweb.net, zemtv.com, dramasonline.com, fashionuniverse.net, tweettunnel.com
Sports and entertainments	twitter.com, youtube.com, facebook.com
Politics	Facebook.com, siasat.pk, twitter.com, youtube.com
Food and recipes	Urduweb.org, facebook.com, friendscorner.com, Pakistan.web.pk, kfoods.com

**Figure 1:**
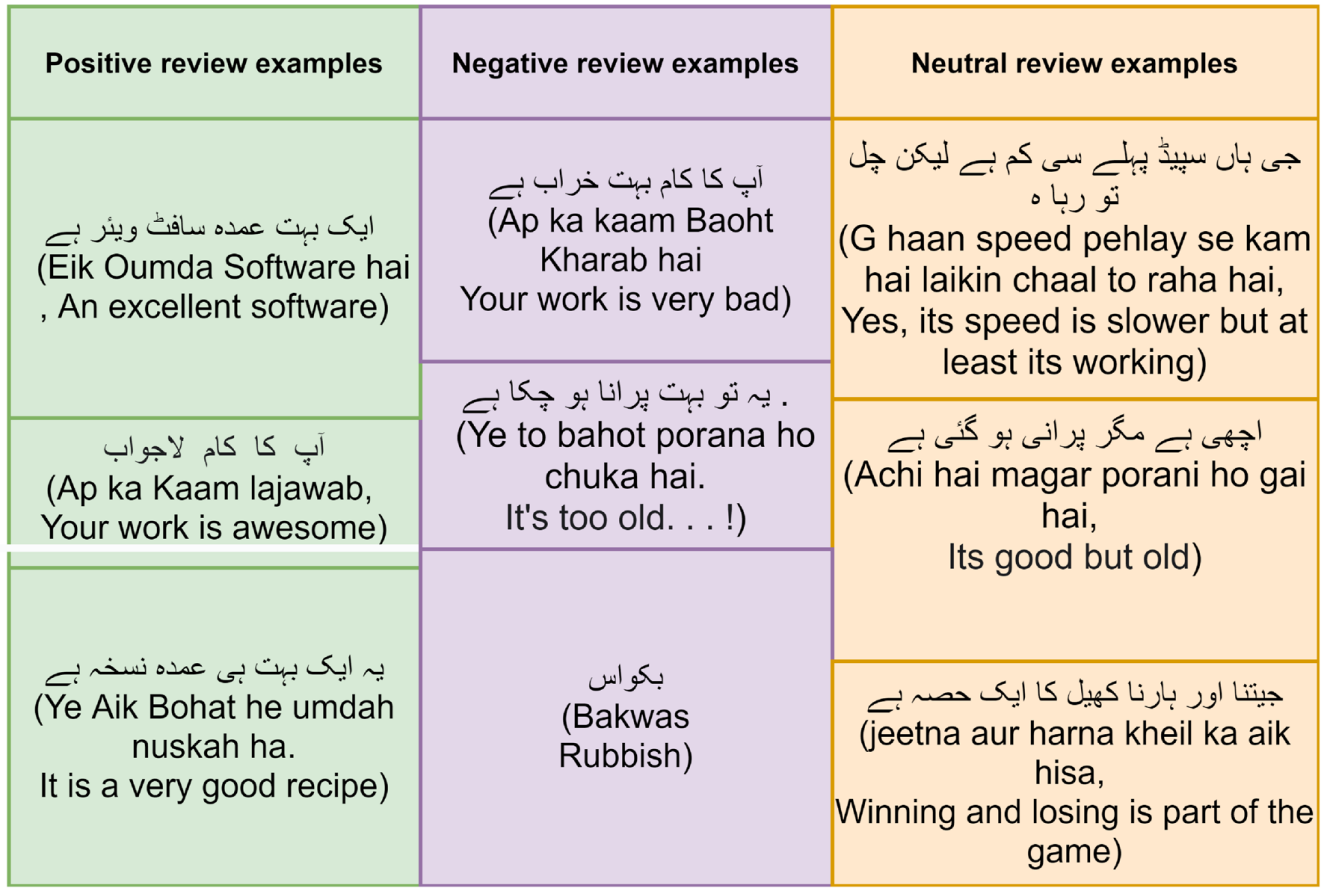
Examples of customer reviews label as neutral, positive and negative.

To implement Urdu SA, we need an annotated corpus containing user comments with their sentiments. Initially, annotations rules were defined then the corpus was annotated manually by three native speakers of the Urdu language keeping in mind those guidelines. All three native Urdu speakers were well aware of the purpose of annotation, annotated the complete dataset. Annotations guidelines were made for Urdu SA from existing literature. Figure 1 shows some samples of comments from the neutral, negative, and positive categories.

### Annotation rules

- A review is considered positive if the specified review expresses a positive meaning for all the characteristic terms. Suppose it contains words such as “acha” good, “Khoubsoorat” beautiful without containing negations like “Na” “Nahi” no as these words change the polarity^55^.
- If any review expressing mutually neutral and positive classes, the review is marked as positive.
- If any review expressing any agreement, then that review is classified as positive^56^.
- If the user review expresses the negative sentiment in all aspects, then the review is marked as negative if it contains terms like “Bora” bad, “bukwas” rubbish, “zolum” cruelness, “ganda” dirty, without containing the negations as negations invert the polarity of the whole sentence^57^.
- If a user comment comprises more negative words than any other class, it is classified as a negative review.
- If a sentence contained straight unsoftened disagreements, then that sentence is classified as negative^56^.
- If a review contained words such as banning, penalizing, assessing, and bidding, then that review is marked as a negative review^56^.
- If a review comprises a denial, then that review is tagged as a negative review.
- If a review contains a negative term with a positive adjective, then that sentence is marked as a negative review^58^.
- Mockery: sentence such as “MashaAllah se koy to rank milli ha na hamari cricket team ko ...akhiri he sahi” (By the grace of God, our cricket team got at least some rank. may that be last) as classified as negative sentences^59^.
- If a sentence contains a question such as “eis team ka kia banay ga” what will happen to this team? Showing frustrations is marked as a negative review^59^.
- If a piece of factual information is presented in a sentence, then the sentence is marked as a neutral sentence?.
- If assumptions, beliefs, or thoughts are shared in a review, then that review is identified as a neutral sentence^60^.
- If words like maybe (Shaid) are present in a review, they are classified as neutral^56^.
- A review containing both negative and positive opinions regarding the aspects is considered a neutral sentence^55^.

### Corpus characteristics

To create the standard corpora, three human experts annotated the whole UCSA-21 dataset. Master graduates annotated each user review; they are native Urdu speakers and are well familiar with SA. To ensure that our annotation guidelines were proper, we gave a random sample of 100 reviews to two annotators (X and Y) and asked them to mark and mention which ones came under which conditions. Individualistically, both annotators classified these sentences into one of three categories: negative, neutral, and positive. The conflicting reviews among annotator x and annotator y were resolved by third annotator z keeping in mind the above-discussed annotations guidelines. For the entire dataset, we achieved an Inter-Annotator Agreement (IAA) of 71.45 percent using Cohens Kappa method. The findings of the IAA score and moderate scores show that the manual annotations rules were adequately drafted, well understood, and followed by annotation specialists during the annotation stage. After evaluating the data, it was shown that the majority of the disagreement occurred between the negative and neutral (11.60%) and positive and neutral (12.01%) classifications. Summary of the corpus presented in Table 3 and 4, the UCSA-21 corpus comprises 9312 Urdu reviews, with 3,422 positive ratings, 2787 negative reviews, and 3103 neutral reviews. The statistics of corpus UCSA-21 show a class balance. Academics have worked hard to create datasets for sentiment analysis studies. Still, most of the available annotated datasets are too small and contain sentences from only a few domains, rather than multiple domains like UCSA-21. The other drawback of most of the existing corpora is they contain only two classes, negative and positive.

**Table 3:** Details of proposed and UCSA Urdu corpus.

Characteristics	Proposed corpus	UCSA corpus
Total number of reviews	9312	9601
Positive reviews	3422	4843
Negative reviews	2787	4758
Neutral reviews	3103	–
Minimum review length in words	1	1
Maximum review length in words	149	–
Total number of tokens	179,791	1,141,716
Average tokens per review	19.30	–

## Proposed methodology

This section contains the experimental description of applied machine learning, rule-based, deep learning algorithms and our proposed two-layer stacked Bi-LSTM model. These algorithms have been trained and tested on our proposed UCSA-21 corpus and UCSA^50^ datasets which are publically available.

**Figure 2:**
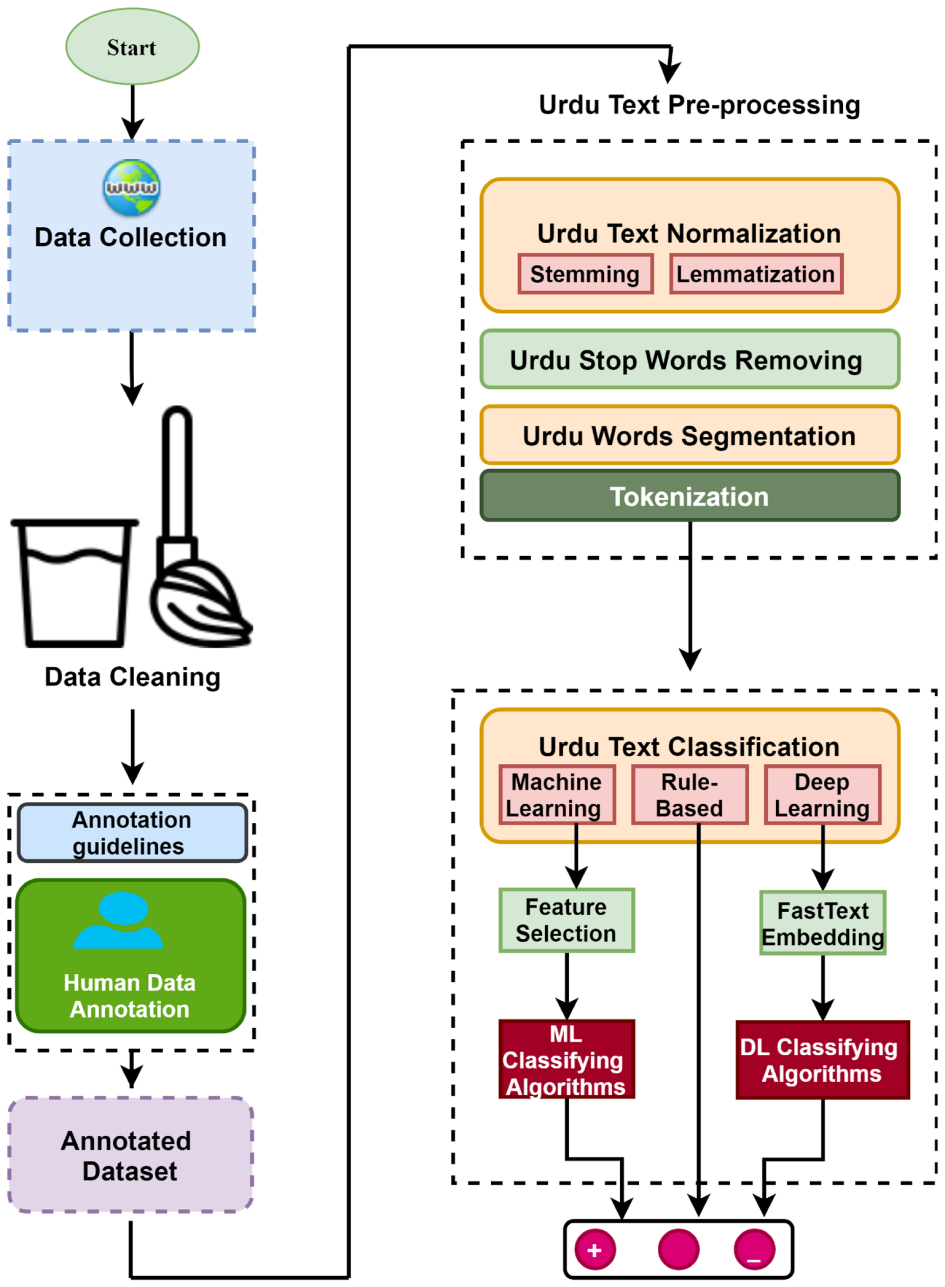
Proposed abstract level architecture for Urdu sentiment analysis.

### Experimental datasets

In this research study, we used two urdu datasets UCSA-21(Our Proposed) and UCSA^50^ to validate our proposed model. The proposed UCSA-21 dataset contains 9,312 Urdu reviews belonging to various genres such as food and recipes, movies, dramas, TV talk shows, politics, software and gadgets, and sports gathered from different social media websites. Each review in UCSA-21 belongs to one of three classes: neutral represented by 0, positive symbolized by 1, and negative reviews represented by 2. Tertiary classifications have experimented on the proposed corpus. The UCSA corpus compromises with total 9601 positive and negative user comments, contains 4843 positive and 4758 negative reviews. Tables 3 and 4 summarized the details of the used datasets in experiments.

**Table 4:** Statistics of proposed dataset.

Genres	Total reviews	Positive reviews	Negative reviews	Neutral reviews
Food and recipes	1250	386	317	547
Movies and drams	1977	590	677	710
Politics	1873	479	744	650
Software and gadgets	2325	1326	455	544
Sports and entertainment	1887	641	594	652
Total	9312	3422	2787	3103

### Pre-processing

The primary goal of pre-processing is to prepare input text for subsequent tasks using various steps such as spelling correction, Urdu text cleaning, tokenization, Urdu word segmentation, normalization of Urdu text, and stop word removal. Tokenization is the process of separating each Uni-gram from sentences. The text is tokenized based on punctuation marks and white spaces. Stop words are vital words of any dialect and have no means in the context of sentiment classifications. They all are removed from the corpus to minimize corpus size. Segmentation is the method to find the boundaries among Urdu words. Due to the morphological structure of the Urdu language, the space between words does not specify a word boundary. Therefore, determining word boundaries in Urdu is essential^41^. Space-omission and Space-insertion are two main issues are linked with Urdu word segmentation. An example of a space omission among two words such as “Alamgeir”, universal and similarly space insertion in a single word such as “Khoub Sorat”, beautiful. In Urdu dialect, many words contain more than one string, such as “Khosh bash,” which means happiness is a Uni-gram with two strings. If during typing, that space between two strings is somehow omitted, then it will become “Khoshbash,” which is wrong syntactically and semantically either.The normalization part can be applied to fix the problem of correct encodings for the Arabic and Urdu characters with appropriate characters. Normalization brings each character in the designated uni-code array (0600-06FF) for the Urdu dialect.

#### Features extraction

Text is often indicated as a vector of weighted features in NLP tasks such as text classification. Different *n*-gram models are utilized in this study; these are models that assign probability to a series of words.A unigram is a model that has a series of one word, such as “Natural”; similarly, a bigram is a sequence of two words, such as “Natural Language,” and a trigram model is a sequence of three words, such as “Natural Language Processing.” On our dataset, we looked at *n*-gram features like unigram, bigram, trigram and variouse combination of these *n*-gram features. Additionally, we also investigate various character gram feattures to gain best results. Recently, pre-trained word embeddings approaches^61^ have experimented with several NLP-related tasks, outperforming the existing systems. The main idea behind these word embedding models is to train them on large amounts of text data and fine-tune them for specific applications. The Wikipedia and Common Crawl (CC) data were used to train the fastText word embedding model. Wikipedia is the biggest free online data source, written in more than 200 dialects. After downloading and cleaning data, the model was trained. CC is a non-profit organization, which crawls web data and makes data freely available. fastText has been trained to understand more than 150 dialects, including Urdu. This is why we choose to use the fastText word vector model in our proposed research. fastText word to vector model was trained using Skipgram^61^ and extension of Continuous Bag of Words (CBOW) methods^61^. In the Skipgram method, word representations are extended with character n-grams. A vector is associated with all n-gram characters, and vectors associated with words are obtained by adding the n-gram characters in the word. Similarly, the CBOW method denotes words as bags of character n-gram.

### Classification techniques

This section explains the details of the proposed set of machine learning, rule-based, a set of deep learning algorithms and proposed mBERT model. The set of machine learning algorithms such as KNN, RF, NB, LR, MLP, SVM, and AdaBoost are used to classify Urdu reviews. Additionally, some deep learning algorithms such as CNN, LSTM, Bi-LSTM, GRU and Bi-GRU with fastText embeddings were also implemented. Figure 2 explains the abstract-level framework from data collection to classification.

#### The rule-based approach

**Figure 3:**
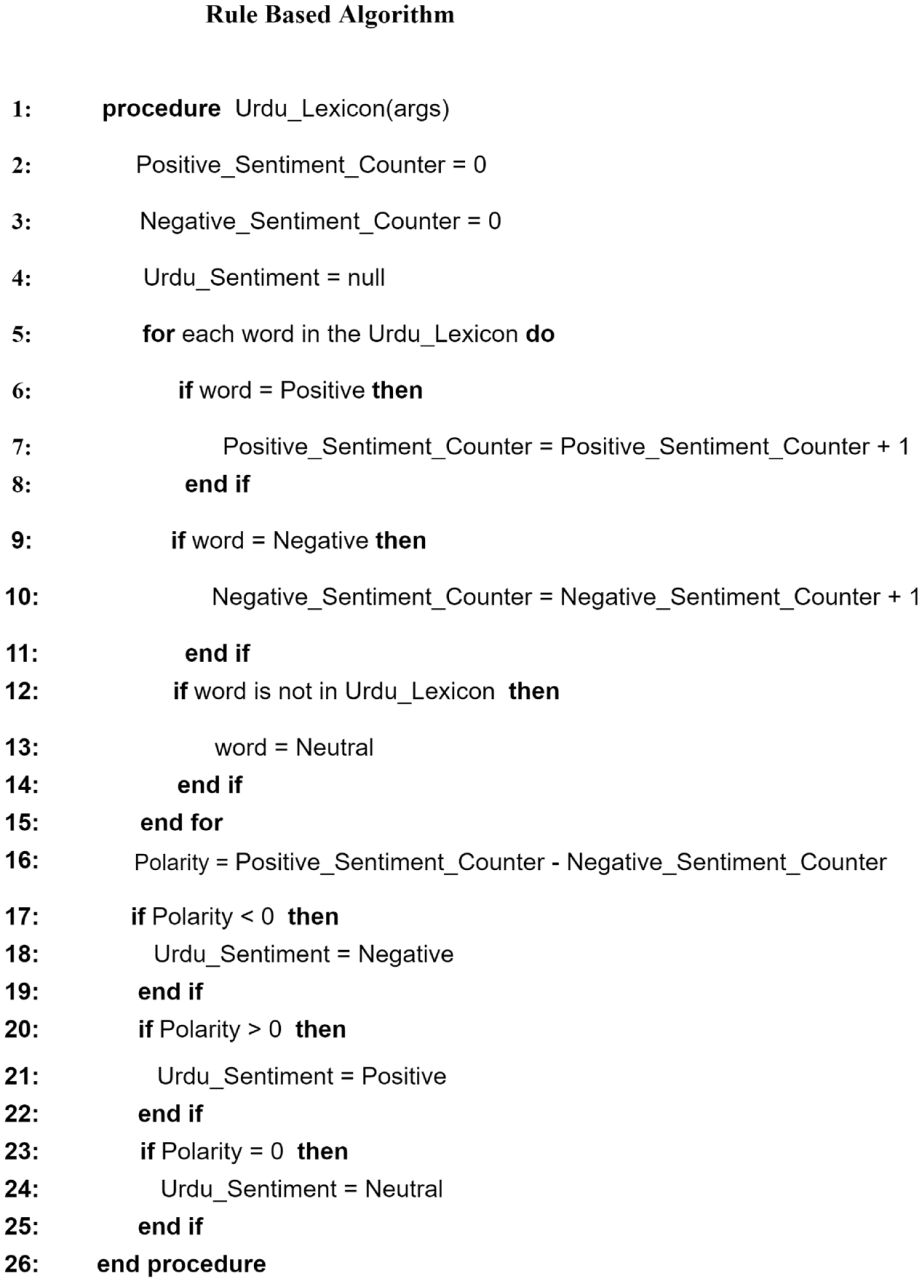
Rule-based Urdu sentiment analysis algorithm using Urdu Lexicon.

Pure Urdu lexicon list containing 4728 negative and 2607 positive opinion words are publicly available. Figure 3 explains the algorithm of this approach in detail. Initially, each sentence is tokenized, and then each token is classified into one of three classes by comparing it to the available opinion words in the Urdu lexicon. The accessible Urdu lexicon and the words are used to determine the overall sentiment of the user review. If the text contains more positive tokens, the review is categorized as positive with a polarity score of 1. A review is characterized as negative with a polarity score of 2 if it contains more negative tokens (words) than positive tokens (words). Finally, a review is defined as neutral with a polarity score of 0 if it contains the same number of negative and positive words.

#### Deep learning models

The deep learning methods such CNN-1D, LSTM, GRU, BI-GRU, Bi-LSTM and mBERT model with word embedding model (fastText) were implemented using keras neural network library 4 for Urdu sentiment analysis to validate our proposed corpus. The technical and experimental information of deep learning algorithms are presented in this section. CNN-1D is mostly utilized in computer vision, but it also excels at classification problems in the natural language processing field. A CNN-1D is particularly capable If you intend to obtain new attributes from brief fixed-length chunks of the entire data set and the position of the feature is irrelevant^62,63^.

Study^64^ introduced GRU to overcome the shortcomings of recurrent neural networks, such as resolving the vanishing gradient problem using update and reset gate mechanisms.Both update and reset gates are essentially vectors that govern what information should be transmitted to the output unit. The most exciting aspect of GRU is that it can be properly trained to keep information for an extended period of time without losing track of timestamps. A sequence processing model with two GRUs is known as Bi-GRU. One takes information in a forward direction, whereas the other takes it backwards. Only the input and forget gates are present in this bidirectional recurrent neural network.

LSTM^65^ is a recurrent neural network design that displays state-of-the-art sequential data findings. LSTM is a technique for capturing long-term dependencies between text data. The LSTM model acquires the current word’s input for each time step, and the prior or last word’s output creates an output, which is utilized to feed to the next state. The prior state’s hidden layer (and, in some cases, all hidden layers) is then used for classification.We use Bi-LSTM model to classify each comment according to its class. Generally, Bi-LSTM used to capture more contextual information from both previous and future time sequences. In this study we used two-layer (Forward and Backward) Bi-LSTM, which obtain word embeddings from FastText.

**Figure 4:**
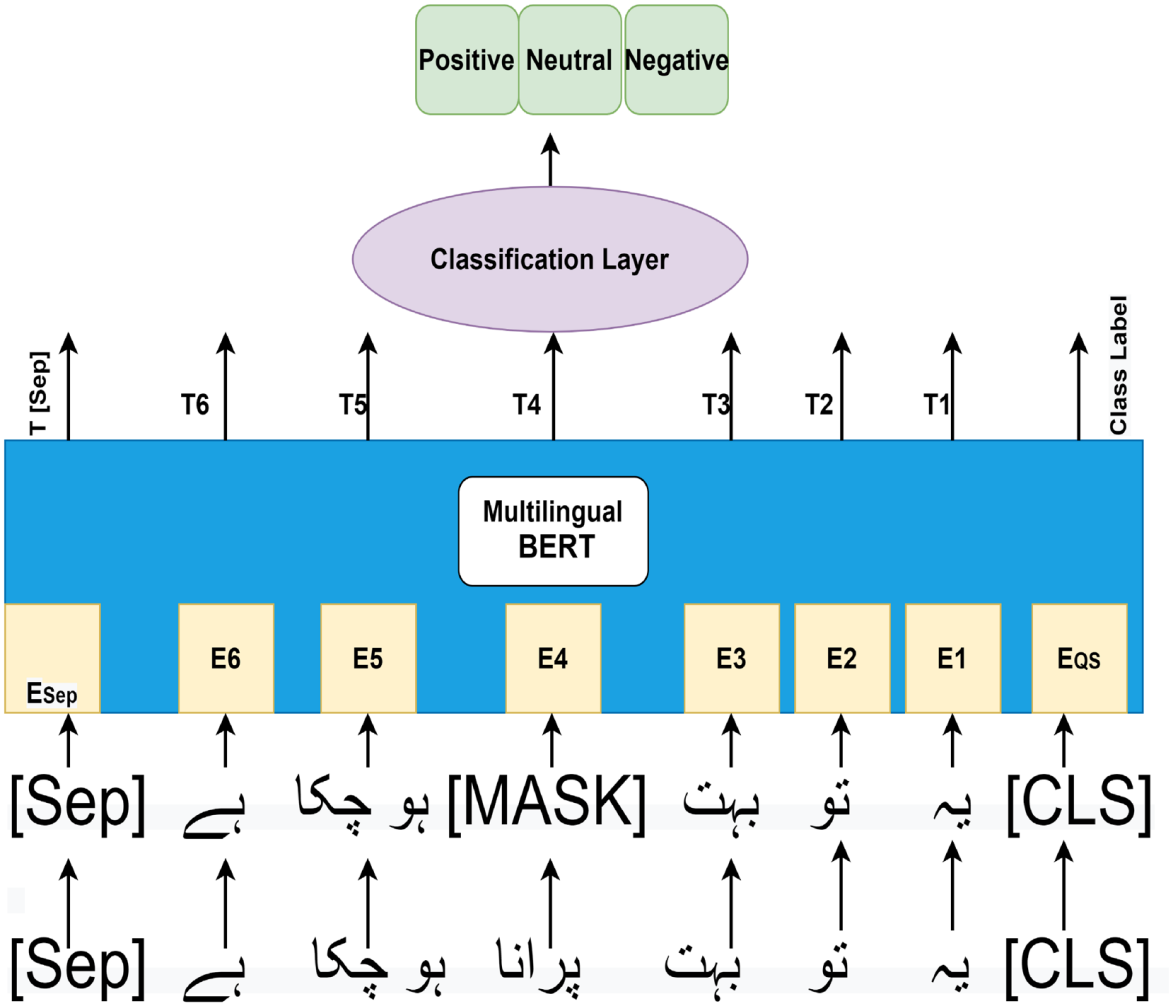
Multilingual BERT high level architecture for Urdu sentiment analysis.

**mBERT:**BERT^66^ is one of the most widely used current language modeling architectures. Its generalization capabilities allows it to be modified to a variety of downstream tasks based on the demands of the user, whether it’s NER or relation extraction, question answering, or sentiment analysis. Figure 4 shows high level architecture of our Proposed model based on Multilingual BERT^67^. We fine-tune the latest multilingual (mBERT) model for Urdu sentiment recognition using supervised training data. The model mBERT developed based on single language base BERT^66^, which consists of 12 transformer layers and 768 hidden layers. The top 104 languages including Urdu with the largest Wikipedias were used to train the mBERT model. The training data for every dialect was gathered from a complete Wikipedia dump (except user and talk pages).

**Transformers:** The BERT small or base has 12 transformer layers, whereas the BERT large has 24 transformer layers. The Transformer is a natural language processing paradigm that aims to do sequence-to-sequence activities with long-range dependencies. The transformers made up with encoders and decoders. Furthermore, an encoder is made up of two pieces. Multi-Head Attention is the first part, while Feed Forward Neural Network is the second part. Masked Multi-Head Attention with Multi-Head Attention Feed Forward Neural Network is also included in Decoder. Encoders and decoders are implemented as stacked on top of each other.

**Attention:** The Transformer relies heavily on attention. Transformers’ self-attention obtains context comprehension of a word in the text based on neighboring words in the sentence. Attention uses Eq. (1) to determine the context of every word.

1 $$\begin{aligned} Attention (Q,K,V)= softmax\left( \frac{QK^T}{\sqrt{d_k}}\right) v \end{aligned}$$

where Q, K, and V are abstract vectors that extract various components from an input word. The special classification token <CLS> in our proposed mBERT model captures the entire sentence, e.g., “Ye tou......” into a fixed-dimensional pooling representation and which produced an output vector with the equal size as the hidden size and the transformers’ output then fed into the fully-connected classification layer, which is the first token’s ultimate hidden state, whereas the special classification token <SEP> indicates the end of this particular sentence, as illustrated in Fig. 4. The second stage is to replace 15% of tokens in each sentence with a [MASK] token (for example, the word ’Porana’ is substituted with a [MASK] token). The context of non-masked tokens is then used by the mBERT model to infer the original values of masked tokens. The encoders assign a unique representation to each token. For instance, the E1 is the fixed presenter of the sentence’s first word, “ye”. The model is made up of many levels, each of which performs multi-headed attention on the output of the preceding layer, for example, mBERT has 12 layers. T1 is the last representation of the first token or word of every sentence in Fig. 4. The classification layer or softmax layer that has been added here. The classification layer has a dimension of K x H, where K is the number of classes (Positive, negative and neutral) and H is the size of the hidden state.

**Model Training and Fine-Tuning:** The entire sentiment classification mBERT model has been trained in two phases, with the first phase involving the pre-training of the mBERT language model and the second phase involving the fine-tuning of the outmost classification layer.The Urdu mBERT has been pre-trained on the Urdu Wikipedia. The mBERT model has been fine-tuned using the training set of the proposed and UCSA datasets, which are Comprised with labelled user reviews. Especially, the fully connected classification layer has been trained in this way. During training, categorical cross-entropy was utilized as the loss function. Table 5 presents  lists the hyper-parameters adopted for this research.

### Evaluation measures

In this study, Urdu sentiment analysis text classification experiments have been performed to evaluate our proposed dataset by using a set of machine learning, rule-based and deep learning algorithms. As a baseline algorithm for better assessment, we performed tertiary classifications experiment with 9312 reviews from our suggested UCSA-21 dataset. We depict four evaluation measures applied for evaluations of a bunch of machine learning, rule-based, and deep learning algorithms such as accuracy, precision, recall, and F1-measure.

$$\begin{aligned} Accuracy= & {} \frac{TP +TN}{TP+TN+FP+FN} \\ Precision= & {} \frac{TP}{TP+FP} \\ Recall= & {} \frac{TP}{TP+FN} \\ F_1\ \ measure= & {} \frac{2\times Precision \times Recall}{Precision+ Recall} \end{aligned}$$

where TN, TP, FN, and FP represent number of True Negative, True Positive, False Negative and False Positive respectively.

## Results analysis

This section explains the results of various experiments that have been executed in this study, the usefulness of our proposed architecture for Urdu SA, and the discussion of revealed results. In the evaluation of various implemented machine learning, deep learning, and rule-based algorithms, it is observed that the mBERT algorithm perform better than all other models.

**Table 5:** mBERT model hyper-parameters.

Hyper-parameter	Value
Learning rate	2e-5
Batch size	16
Number of epochs	15
Attention heads	12
Gradient accumulation steps	16
Hidden size	768
Hidden layers	12
Maximum sequence length	128
Parameters	110 M

**Table 6:** Urdu sentiment analysis results using machine learning models with word *n*-gram features.

Feature	Model	Accuracy	Precision	Recall	F1 Score
Unigram	KNN	67.23	63.31	70.34	66.64
	RF	65.80	62.07	69.12	65.40
	NB	68.70	65.45	70.19	67.73
	LR	64.70	61.90	67.01	64.35
	MLP	67.81	65.01	70.22	67.46
	SVM	71.66	69.02	72.76	70.84
	AdaBoost	69.23	66.99	71.01	68.94
Bigram	KNN	61.73	59.21	63.04	61.06
	RF	60.58	58.97	62.10	60.49
	NB	64.39	62.05	66.20	64.05
	LR	60.24	58.10	61.98	59.97
	MLP	63.30	60.01	65.02	62.28
	SVM	67.96	64.45	69.00	66.64
	AdaBoost	64.03	61.90	66.10	63.93
Trigram	KNN	58.13	48.88	68.04	57.19
	RF	55.39	47.00	67.20	55.31
	NB	59.20	51.05	70.20	59.11
	LR	55.00	47.09	65.80	54.89
	MLP	57.40	49.10	68.78	57.29
	SVM	61.66	50.00	68.10	61.25
	AdaBoost	58.50	51.01	67.80	58.21
Combination (1–2)	KNN	67.62	66.02	69.30	67.62
	RF	66.95	65.07	68.89	66.92
	NB	70.10	68.06	71.97	69.96
	LR	66.30	64.16	67.32	65.70
	MLP	69.91	67.23	70.98	69.05
	SVM	72.71	71.05	74.10	72.54
	AdaBoost	70.60	69.00	72.11	70.52
Combination (1–3)	KNN	67.80	66.80	68.33	67.55
	RF	66.70	65.70	67.32	66.50
	NB	69.50	68.44	70.12	69.26
	LR	66.00	64.70	66.39	65.53
	MLP	69.80	68.09	70.30	69.17
	SVM	71.30	70.30	72.20	71.23
	AdaBoost	71.00	69.70	71.59	70.63

Tables 6 and 7 presents the obtained results using various machine learning techniques with different features on our proposed UCSA-21 corpus. The results reveal that SVM performance is slightly better on the UCSA-21 dataset than other machine learning algorithms, with an accuracy of 72.71% using combination (1-2) features. The gained results clearly show that all the machine learning classifiers perform better with word feature combination (1-2) and unigram. On the other hand, obtained results indicating that the set of machine learning algorithms performance is not satisfiable with trigram and bigram word feature. RF gain 55.00 % accuracy using trigram features had the lowest accuracy of all machine learning classifiers. When compared to bigram and trigram word features, all machine learning classifiers perform better using unigram word features which is consistent with^50^.The outcomes of several machine learning methods using character gram features are represented in Table 7. Using the Char-3-gram feature, the findings demonstrated that NB and SVM outperformed all other machine learning classifiers with an accuracy of 68.29% and 67.50% respectively. on the other hand, LR had the poorest performance, with an accuracy of 58.40% when employing the char-5-gram feature.

**Table 7:** Urdu sentiment analysis results using machine learning models with char *n*-gram features.

Feature	Model	Accuracy	Precision	Recall	F1 Score
Char-3-Gram	KNN	65.23	61.31	68.34	64.63
	RF	64.70	61.07	67.12	63.95
	NB	68.29	63.45	70.19	66.65
	LR	64.60	62.90	66.01	64.41
	MLP	66.71	63.01	68.22	65.51
	SVM	67.50	64.02	68.76	66.30
	AdaBoost	64.90	62.99	66.01	64.66
Char-4-Gram	KNN	60.75	59.21	62.04	60.59
	RF	60.30	57.97	60.10	59.01
	NB	63.40	60.05	64.20	62.05
	LR	60.24	57.10	60.98	58.98
	MLP	62.10	58.15	64.10	60.98
	SVM	65.90	62.35	67.10	64.63
	AdaBoost	62.90	60.70	64.20	62.40
Char-5-Gram	KNN	60.00	58.10	61.10	59.56
	RF	58.70	56.90	59.00	57.93
	NB	62.46	59.05	62.10	60.53
	LR	58.40	55.10	59.90	57.39
	MLP	60.10	56.01	62.00	58.85
	SVM	63.55	60.45	64.10	62.22
	AdaBoost	61.00	59.60	61.00	60.29

Table 8 presents the baseline results achieved using a rule-based approach to validate our proposed UCSA-21 dataset. The rule-based approach achieved an accuracy (64.20%), precision (60.50%), recall (68.09%), and F1 score (64.07. It is observed that the rule-based technique didn’t achieve high scores in terms of accuracy as compared to machine learning and deep learning approaches. The lousy performance of the rule-based approach in this experiment is mere because of not considering the semantic information during the experiment; the experiment is only based on the terms in the lexicons database. One of the biggest flaws with rule-based algorithms is that it cannot distinguish humorous reviews with more positive words.The satirical reviews such as “MashaAllah se koy to rank milli ha na hamari cricket team ko. . . akhiri he sahi” translated as “By the grace of God, our cricket team got at least some rank. may that be last)” is a negative review which is wrongly classified as a positive review by rule-based approach.

**Table 8:** Urdu sentiment analysis results using rule-based algorithm.

Model	Accuracy	Precision	Recall	F1 Score
Rule-based	64.20	60.50	68.09	64.07

**Table 9:** Urdu sentiment analysis results using deep learning models for UCSA-21 Corpus.

Word Embedding	Model	Accuracy	Precision	Recall	F1 Score
fastText	Bi-LSTM	76.50	75.01	77.14	76.06
	Bi-GRU	75.60	73.10	76.70	74.85
	CNN-1D	72.10	69.79	72.70	71.21
	CNN-1D+MP	70.09	68.79	70.70	69.73
	CNN-1D+ATT	73.80	71.79	75.70	73.69
	LSTM	73.15	71.40	74.28	72.49
	LSTM+MP	72.15	70.40	73.28	71.81
	LSTM+ATT	74.80	72.40	76.28	74.41
	GRU	72.50	71.00	72.00	71.49
BERT	Proposed model	77.61	76.15	78.25	77.18

**Table 10:** Urdu sentiment analysis results using deep learning models for UCSA corpus.

Word Embedding	Model	Accuracy	Precision	Recall	F1 Score
fastText	Bi-LSTM	81.10	80.20	80.55	80.37
	Bi-GRU	80.55	80.05	80.15	80.09
	CNN-1D	78.10	78.43	76.78	77.59
	CNN-1D+MP	77.60	77.05	75.25	76.13
	CNN-1D+ATT	79.05	78.00	7.45	78.15
	LSTM	78.85	77.76	77.83	77.79
	LSTM+MP	77.55	76.50	76.45	76.47
	LSTM+ATT	79.05	79.80	78.50	78.67
	GRU	78.35	77.30	77.15	77.22
BERT	Proposed model	82.50	81.35	81.65	81.49

Finally, this section contains the baseline results generated using many deep learning algorithms such as CNN-1D, LSTM,GRU, Bi-GRU, Bi-LSTM and our proposed model based on mBERT model. According to the results presented in Table 9, deep learning models outperforms machine learning and rule-based approach. The obtained results reveal that our proposed model fine-tuned based on mBERT with SoftMax supersedes all other deep learning models with accuracy, precision, recall, and F1 score of 77.61%, 76.15%, 78.25%, and 77.18% respectively. It is Observed that Bi-LSTM and Bi-GRU can be effective for Urdu sentiment analysis compared to other traditional machine learning, rule-based, and deep learning algorithms merely because Bi-LSTM and Bi-GRU can capture information from backward and forward ways. Bi-LSTM produces slightly better results because it understands context better than LSTM and CNN-1D. It is also observed that LSTM and CNN-1D achieves slightly better results with Attention (ATT)layer as compared Max-polling (MP) layer.

**Figure 5:**
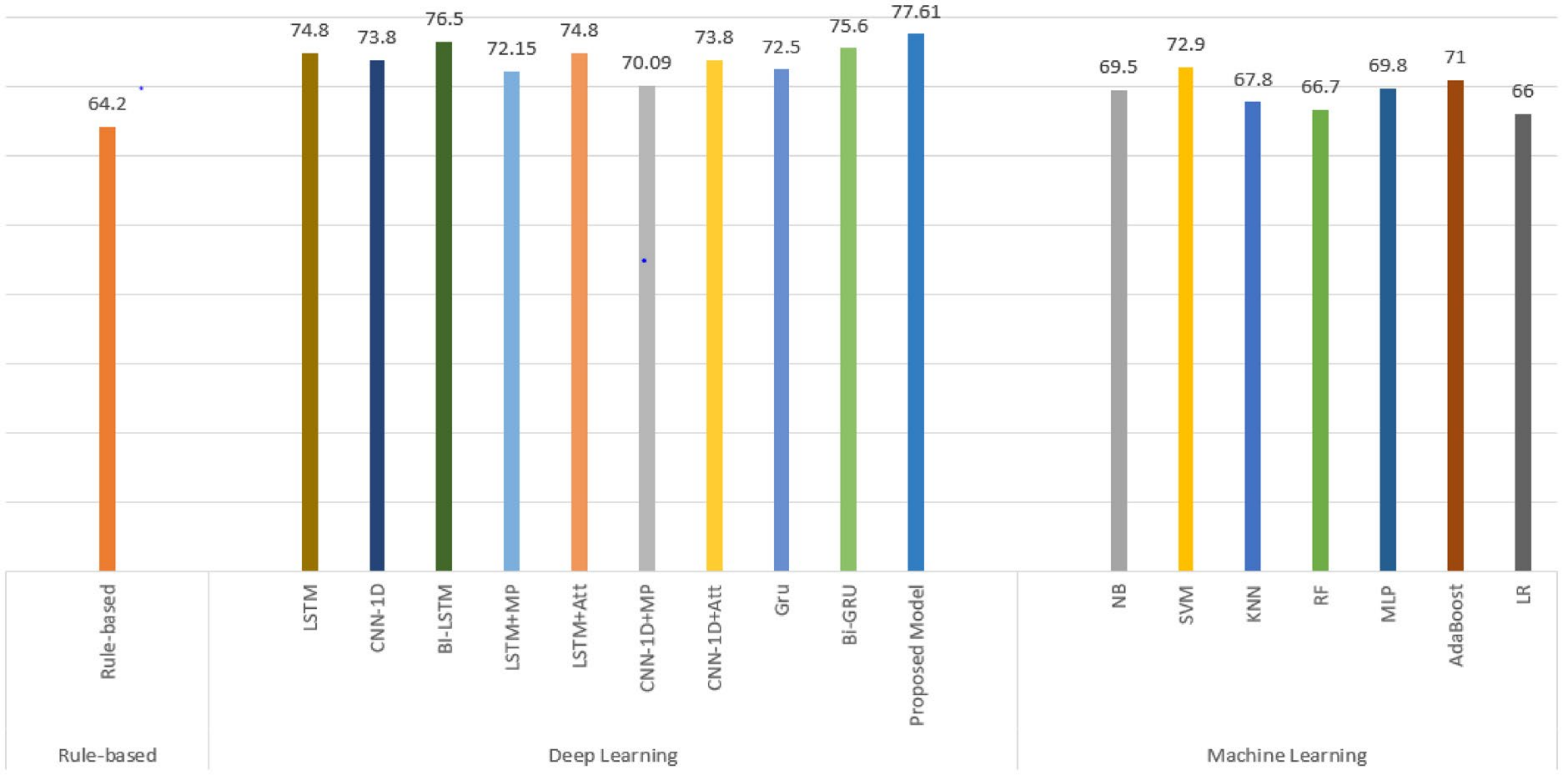
Accuracy Comparison of Machine, Deep Learning and Rule-Based Approaches with Proposed Model using UCSA-21 Corpus.

Using the UCSA corpus, Table 10 compares the results of our proposed mBERT model with those of other commonly used deep learning algorithms. The obtained results shows that mBERT with SoftMax outperform all other deep learning algorithms with accuracy, precision, recall, and F1 score of 82.50%, 81.35%, 81.65%, and 81.49% respectively.We did not apply traditional machine learning algorithms to validate UCSA corpus because in study^50^ authors already set baseline results. The findings shows that deep learning and our proposed model comparatively perform better by using UCSA corpus, due to less number of classification classes. As mentioned above the UCSA corpus compromises with only two classes: Positive and Negative on the other hand our proposed UCSA-21 corpus comprises with additional neutral class. After evaluating the data, achieving highest performance on both datasets shows the effectiveness of our proposed model for Urdu sentiment analysis (Fig. 5).

**Figure 6:**
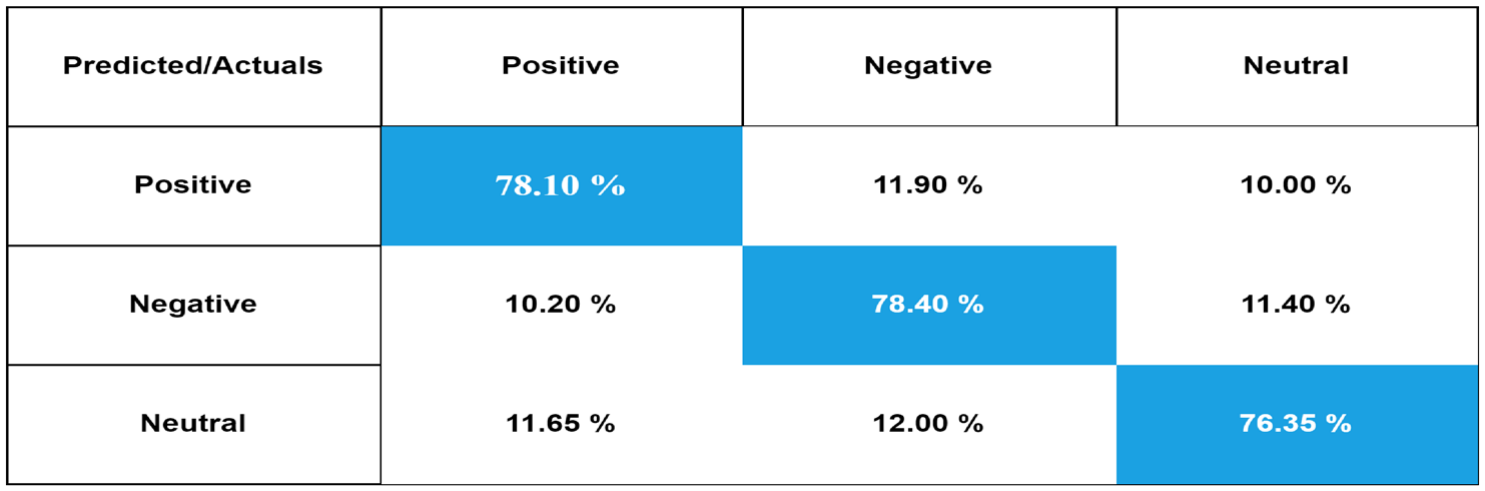
Confusion matrix of our proposed model using our proposed UCSA-21 corpus.

**Figure 7:**
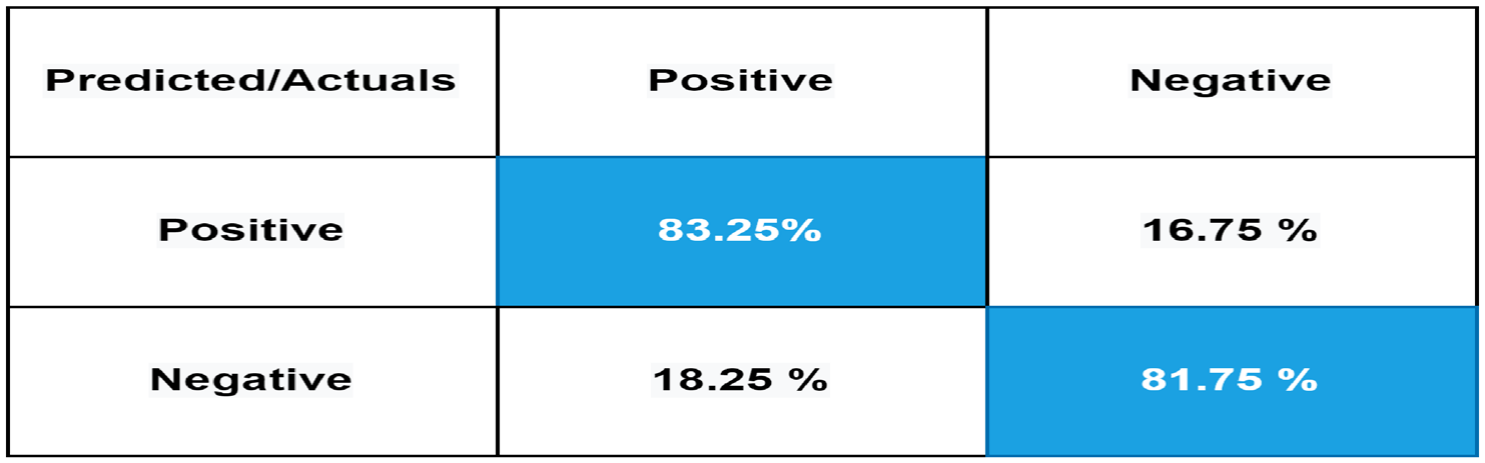
Confusion matrix of our proposed model using UCSA corpus.

The confusion matrix is a measure for assessing the validity of a classification. Figure 6 present the confusion matrix of our proposed mBERT by using UCSA-21 Urdu corpus. In Fig. 6, 78.10% of positive sentences are correctly classified as positive, while only 11.90% of positive reviews are incorrectly classified as negative, and 10.00% as neutral. Out of all reviews 78.40% of negative reviews are correctly identified as negative, while only 11.40% and 10.20% of negative reviews are incorrectly classified as neutral and positive respectively. Only 12.00% and 11.65% of neutral reviews are misclassified as negative and positive respectively, while 76.35 % of neutral reviews are accurately classified by our proposed model against UCSA-21 corpus. Similarly, Fig. 7 represents the confusion matrix of our proposed mBERT model using UCSA corpus which has only two classes: positive and Negative.

Machine learning models, on average, contain less trainable parameters than deep neural networks, which explains why they train so quickly. Instead than employing semantic information, these classifiers define class boundaries based on the discriminative power of words in relation to their classes. Furthermore, SVM performs pretty well among all adopted machine learning approaches because it not only handles outliers significantly better than other machine learning algorithms by deriving maximum margin hyperplanes, However, it also supports the kernel technique, which allows for effective tuning of a number of hyper-parameters to reach optimal performance. In addition, SVM employs Hinge loss, which outperforms LR’s log loss. Similarly, SVM’s capacity to capture feature interactions to some extent makes it superior to NB, which typically treats features independently.

On the other hand, deep learning algorithms, not only automate the feature engineering process, but they are also significantly more capable of extracting hidden patterns than machine learning classifiers. Due to a lack of training data, machine learning approaches are invariably less successful than deep learning algorithms. This is exactly the situation with the hand-on Urdu sentiment analysis assignment, where proposed and customized deep learning approaches significantly outperform machine learning methodologies. Bi-LSTM and Bi-Gru are the adaptable deep learning approach that can capture information in both backward and forward directions. The proposed mBERT used BERT word vector representation which is highly effectiv for NLP tasks. Eventually this approach which is based on transformers and encoder-decoder based technology beats other deep learning, machine learning and rule-based models. Figure 5 compare the overall accuracy of three various approaches and with proposed model used for Urdu sentiment analysis. The results reveals that the proposed mBERT model beats the deep learning, machine learning and rule-based algorithms.

As previously said, the Urdu language has a morphological structure that is highly unique, exceedingly rich, and complex when compared to other resource-rich languages. Urdu is a blend of several languages, including Hindi, Arabic, Turkish, Persian, and Sanskrit, and contains loan words from these languages. These are the most common causes of algorithm misclassifications. Other reasons for incorrect classifications include the fact that the normalization of Urdu text is not yet perfect. To tokenize Urdu text, spaces between words must be removed/inserted because the boundary between words is not visibly apparent. Similarly, in an Urdu sentence, the order of words can be changed but the sense/meaning stays the same, as in “Meeithay aam hain” and “Aam meeithay hain,” both of which have the same meaning “Mangos are sweet”. Manual annotation of user reviews also one of the reasons for miss classification.

The primary purpose for using a set of machine learning algorithms with word and character n-gram features to establish baseline results against our proposed Urdu corpus. Our proposed dataset comprises with short and long type of user reviews that’s why we used various deep learning algroithms such GRU and LSTM to investigate the performance of algroithms against Urdu text. GRU is typically used to categorize short sentences, whereas LSTM is thought to perform better versus long sentences because to its core structure. Similarly, BERT is currently one of the highest performing models for unsupervised pre-training. To address the Masked Language Modelling objective, this model is based on the Transformer architecture and trained on a huge amount of unlabeled texts from Wikipedia. It shows outstanding performance on a variety of NLP tasks. Motivation using mBERT is to investigate its performance against resource deprived languages such as Urdu.

As previously stated, there is a paucity of research on using deep learning approaches to analyze Urdu sentiment. Only a few studies have been published in this field, and they all used various machine learning classifiers on a small dataset with limited domains and have only positive and negative classes. On the other hand, our dataset, contains more user reviews than earlier studies, and it includes several genres with three classifications classes: positive, negative, and neutral. Table 1 shows a summery and comparison of our research with previous research.

## Conclusion and implications

A huge amount of data has been generated on social media platforms, which contains crucial information for various applications. As a result, sentiment analysis is critical for analyzing public perceptions of any product or service. We observed that in the Urdu language, majority of studies focused on language processing tasks, with only a few experiments done in the domain of Urdu sentiment analysis utilizing several classical machine learning methodologies relatively with a small data corpus with only two data classes. In contrast, we proposed a multi-class Urdu sentiment analysis dataset and used various machine and deep learning algorithms to create baseline results. Additionally, our proposed mBERT classifier, achieves F1 score of 81.49% and 77.18% using UCSA and UCSA-21 datasets respectively.

This paper lays the path for more deep learning research into constructing language-independent models for languages with limited resources. Our findings reveal an essential insight: deep learning with pre-trained word embedding is a viable strategy for dealing with complicated and resource-poor languages like Urdu. In future, our plan is to use models such as GPT, GPT2 and GPT3 to improve the results. We believe that our publicly available dataset will serve as a baseline for sentiment analysis in Urdu.
